# Ability to Utilize Digital Health Services: Validation of the Digital HealthCare Scale in Adolescents and Young Adults

**DOI:** 10.3928/24748307-20241204-01

**Published:** 2025-01

**Authors:** Christopher Le, Hanne Søberg Finbråten, Robert Griebler, Diane Levin-Zamir, Øystein Guttersrud

## Abstract

**Background:**

While adolescents and young adults are increasingly expected to take more responsibility for their health and wellbeing, continuing digital transformation and increased implementation of digital health services (DHS) demand skills to utilize digital solutions offered to successfully undertake self-care and self-management. However, research is lacking regarding measurement of adolescents' and young adults' “ability to utilize DHS” (or “DHC”).

**Objective:**

This study aims to measure young people's DHC by (1) validating the Digital HealthCare Scale (DHC scale) in adolescents and in young adults and (2) exploring the extent to which DHC and digital health literacy (DHL) are associated with the number of general practitioner, emergency, or specialist visits.

**Methods:**

A cross-sectional survey was conducted among 890 Norwegian adolescents and young adults age 16 to 25 years. Data were collected from April 2020 to October 2020 using computer-assisted telephone interviewing. Rasch modeling, independent samples *t*-test, chi-square test, and negative binomial regression models were used to analyze the data.

**Key Results:**

The DHC scale is considered valid for measuring DHC in adolescents and young adults, showing sufficient unidimensionality, good overall data-model fit, and no disordered response categories nor differential item functioning. Results showed that female participants and adolescents age 16 to 20 years self-reported significantly lower DHL and DHC than male participants and young adults age 21 to 25 years. Regression analyses displayed a statistically significant association between adolescents' and young adults' DHL (*n* = 371) and DHC (*n* = 389) and their utilization of specialist health services. For every unit (logit) increase in DHL and DHC, the number of specialist visits decreased by 25% and 28%, respectively.

**Conclusions:**

Aligned with previous research calling for new up-to-date instruments to measure the new aspects of DHL, our study has introduced a new measurement scale (DHC scale) for use among adolescents and young adults. This scale may be useful for health authorities, public health workers, and health providers in evaluating and adapting DHC. [***HLRP: Health Literacy Research and Practice*. 2025;9(1):e19–e28.**]

## Abstract (plain language summary)

**Plain Language Summary: **We validated the DHC scale for measuring young people's ability to use DHS. We found that DHL and DHC differed between sex and age groups and associated with the number of specialist visits. Findings foster a forward-thinking approach to measure and promote young people's health literacy toward the digitally transitioning generation.

Technologies solves challenges and provides new opportunities but may also create new dilemmas (Borges do Nascimento et al., 2023; Smith & Magnani, 2019). Digital health services (DHS) are a useful complement to in-person health care, and new technologies alter practices, mindsets, and the way society solves tasks and challenges (Løberg, 2021). In line with the digitalization of society, the health services are also becoming increasingly digital (Levin-Zamir & Bertschi, 2018). However, digitalization is not fully implemented in clinical practice or fully accepted by the public, as many barriers and “digital divides” remain (Koopman et al., 2014; United Nations, 2020).

The literature has suggested that most young people are using the internet as a valid resource to obtain health information (Beck et al., 2014; Park & Kwon, 2018). Those who were born into the digital era seem to prefer seeking out health information electronically more than so-called “digital immigrants” (Haluza et al., 2017; Keles et al., 2020). Although young people are exposed to a wealth of health information (Haugen et al., 2022), their ability to find, access, understand, critically appraise, and apply such health information remains insufficient (Riiser et al., 2020).

A common aspect of all digital technologies is that it requires people to possess general digital skills (Fagnano et al., 2012). Consequently, when using DHS, people need a range of skills such as eHealth or digital health literacy (DHL), which covers the ability to seek, find, understand, appraise, and apply health information from digital sources to addressing or solving a health problem (Kayser et al., 2018; Le et al., 2024; Levin-Zamir et al., 2021; Norman & Skinner, 2006).

Already in 2001, eHealth was defined as “health services and information delivered or enhanced through the internet and related technologies” (Eysenbach, 2001, p. 1) and has since been used interchangeably with another term: “digital health” (Levin-Zamir et al., 2021). Generically, “ability to utilize DHS” (or “DHC”) was suggested to include communication between health care providers and service users through electronic devices (including video, image and text processing), digital health records, digital patient education, digital health portals, and other digital applications for patients, and relatives and/or caregivers (Neupert & Mundie, 2009). The continuing digital transformation and increasing magnitude of DHS fosters higher expectations for and demands on people's self-care and self-management (Fagnano et al., 2012). Given the patient-centered approach to health care, it is paramount that patients, their relatives, and/or caregivers have the ability to best manage their personal health through digital services and channels. DHC refers to the skills needed for being able to use digital tools to follow-up on an individual's own health and illness (Le et al., 2021).

For measuring the type of skills covering the ability to process digital health information, there have been previous instrument developments such as the HLS_19_-DIGI instrument, which was currently validated for use among adolescents and young adults (Le et al., 2024). The other tool, Digital HealthCare Scale (DHC scale), measuring the ability to use DHS was developed within the Norwegian part of the M-POHLs (World Health Organization [WHO] Europe Action Network on Measuring Population and Organizational Health Literacy) first project—The Health Literacy Survey 2019 to 2021 (HLS_19_) (Austrian National Public Health Institute, 2021; Le et al., 2021). This tool was designed to conceptually compliment the HLS_19_-DIGI instrument within the overall DHL module and refers to the specific dimension in which people's ability to use DHS is addressed. This perspective and aspect of the overall DHL concept was recently identified as a knowledge gap in research (Faux-Nightingale et al., 2022).

Hence, the aim of this study was to measure the ability to use DHS in adolescents and young adults by (1) validating the DHC scale in young people and (2) by exploring the associations between DHL/DHC with the extent to which adolescents and young adults were using health care services in terms of general practitioner (GP), emergency, and specialist health care service visits.

While the United Nations defines “youth” as those between ages 15 and 24 years (United Nations, 2024), the World Health Organization defines “adolescents” as those between ages 10 and 19 years (World Health Organization, 2024). Additionally, the terms “youth” and “young people” are used interchangeably for the same age group, which is used without prejudice to other definitions by Member states. In Norway, many health clinics for adolescents provide services to young people age 13 to 25 years (Bergen Municipality, 2024). In our study, data were collected from people age 16 years and older. We referred to the population that was studied as “youth” and used it interchangeably with “young people,” young adults, and adolescents.

## Methods

### Sampling and Data Collection

The HLS_19_ study provided the data that was collected from April 2020 to October 2020. Data were collected using computer-assisted telephone interviewing, and the data collection was stratified by 8 age groups, 2 sexes, and 11 counties, of which this study's sample included 2 age groups (age 16 to 17 years and age 18 to 25 years). Of the 6,000 participants, 890 met our inclusion criteria—adolescents and young adults age 16 to 25 years. The inclusion criteria was mainly based on the fact that many of the compulsory health clinics for adolescents in Norway provide services to users age 13 to 25 years. Due to a differentiated data collection as described in Le et al. (2021, 2022), the DHC scale obtained a smaller sample size (*n* = 471).

In addition to data based on the HLS_19_-DIGI and DHC scales, this study also used sociodemographic factors such as age, sex, education, self-perceived social status, and self-reported financial deprivation, as well as health service utilization variables by number of emergency, GP, and specialist visits. While the sociodemographic factors were included due to their mainstreaming status as determinants of health, the health service utilization variables were selected in light of the Vienna model of health literacy (The HLS_19_ Consortium of the WHO Action Network M-POHL, 2021). Health service utilization in terms of “illness behavior” is one of the factors in the Vienna model, which can be influenced by individual health literacy.

### Measures

The DHC scale was developed building on the competence needed to use DHS, which involves people's knowledge, motivation, and skills. First, the scale consists of seven items covering the motivation to engage in one's own health that strengthens a positive attitude toward the use of DHS. Second, it assesses the extent to which people are capable of using electronic devices, which is necessary to possess a feeling of safety and control when using DHS. Third, the scale takes into consideration the extent to which people are knowledgeable, which is necessary to proceed a meaningful interpretation of health messages and understanding the state of their health.

The DHC scale and the HLS_19_-DIGI scale both use a 4-point rating scale with the response categories as follows: (1) *very difficult*, (2) *difficult*, (3) *easy*, and (4) *very easy*. As the data collection used computer-assisted telephone interviewing, the *don't know* response category was only used when stated spontaneously by the participants. This category was, however, recoded as missing in the data analyses.

For the sociodemographic factors and health care utilization measures, a detailed description of how the data were measured and categorized is provided in **Table A**. However, all sociodemographic factors were dichotomized in the statistical analyses: age (1:16 to 20; 2:21 to 25); education (1: upper secondary education or below; 2: above upper secondary education); self-perceived social status (1: low = level 1 to 5; 2: high = level 6 to 10); and financial deprivation (1: yes; 2: no).

**Table A.**
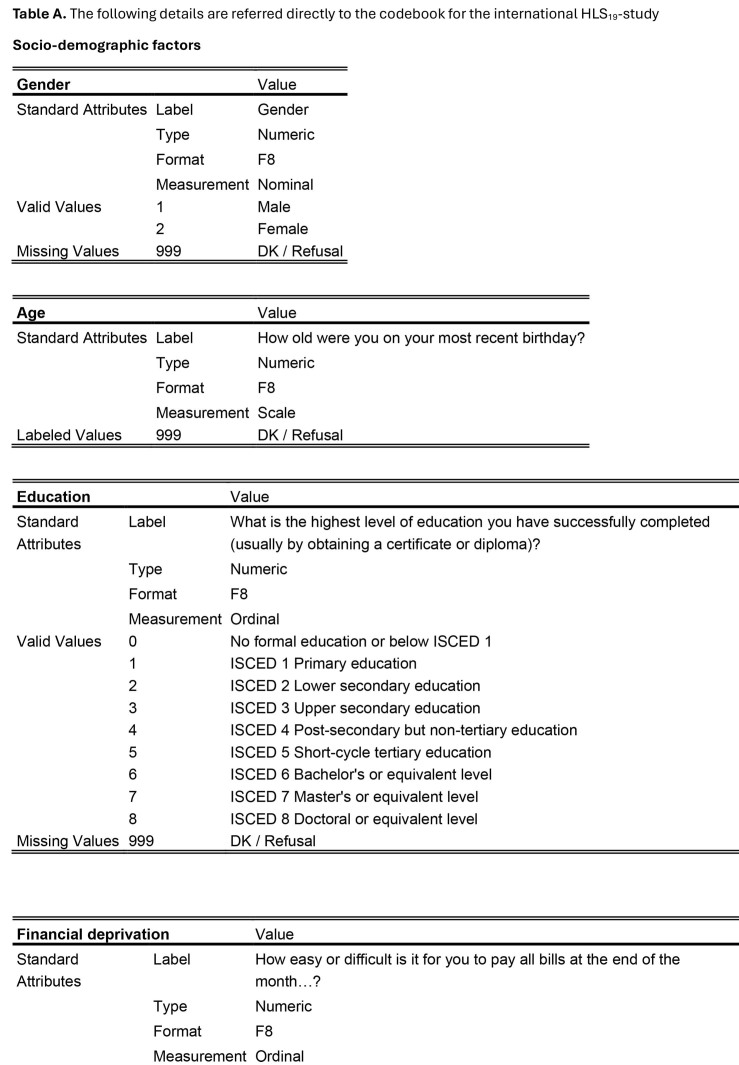
The following details are referred directly to the codebook for the international HLS_19_-study Socio-demographic factors

**Gender**		Value
Standard Attributes	Label	Gender
	Type	Numeric
	Format	F8
	Measurement	Nominal
Valid Values	1	Male
	2	Female
Missing Values	999	DK / Refusal

**Age**		Value
Standard Attributes	Label	How old were you on your most recent birthday?
	Type	Numeric
	Format	F8
	Measurement	Scale
Labeled Values	999	DK / Refusal

**Education**		Value
Standard Attributes	Label	What is the highest level of education you have successfully completed (usually by obtaining a certificate or diploma)?
	Type	Numeric
	Format	F8
	Measurement	Ordinal
Valid Values	0	No formal education or below ISCED 1
	1	ISCED 1 Primary education
	2	ISCED 2 Lower secondary education
	3	ISCED 3 Upper secondary education
	4	ISCED 4 Post-secondary but non-tertiary education
	5	ISCED 5 Short-cycle tertiary education
	6	ISCED 6 Bachelor's or equivalent level
	7	ISCED 7 Master's or equivalent level
	8	ISCED 8 Doctoral or equivalent level
Missing Values	999	DK / Refusal

**Financial deprivation**		Value
Standard Attributes	Label	How easy or difficult is it for you to pay all bills at the end of the month…?
	Type	Numeric
	Format	F8
	Measurement	Ordinal

**Table:**
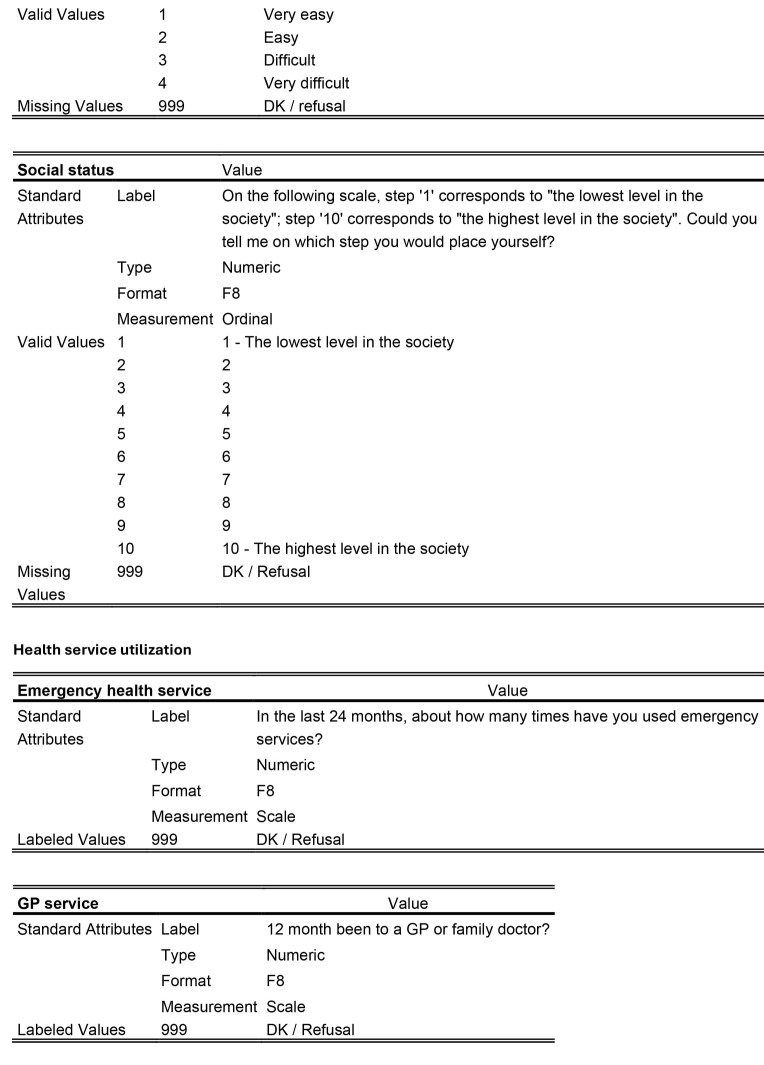


Valid Values	1	Very easy
	2	Easy
	3	Difficult
	4	Very difficult
Missing Values	999	DK / refusal

**Social status**		Value
Standard Attributes	Label	On the following scale, step ‘1’ corresponds to “the lowest level in the society”; step ‘10’ corresponds to “the highest level in the society”. Could you tell me on which step you would place yourself?
	Type	Numeric
	Format	F8
	Measurement	Ordinal
Valid Values	1	1 - The lowest level in the society
	2	2
	3	3
	4	4
	5	5
	6	6
	7	7
	8	8
	9	9
	10	10 - The highest level in the society
Missing	999	DK / Refusal
Values		

**Health service utilization**		

**Emergency health service**		Value
Standard Attributes	Label	In the last 24 months, about how many times have you used emergency services?
	Type	Numeric
	Format	F8
	Measurement	Scale
Labeled Values	999	DK / Refusal

**GP service**		Value
Standard Attributes	Label	12 month been to a GP or family doctor?
	Type	Numeric
	Format	F8
	Measurement	Scale
Labeled Values	999	DK / Refusal

**Table:**
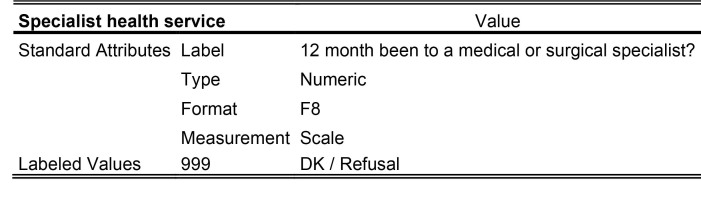


**Specialist health service**		Value
Standard Attributes	Label	12 month been to a medical or surgical specialist?
	Type	Numeric
	Format	F8
	Measurement	Scale
Labeled Values	999	DK / Refusal

### Statistical Analysis

The psychometric properties of the DHC scale were assessed using Rasch modeling, including dimensionality, response dependency, targeting, reliability, item fit, differential item functioning, and ordering of response categories. The Rasch analysis methodology is thoroughly described in Le et al. (2022). Alongside this description, response dependency in this study is indicated by a more commonly used cut-off set by residual correlations above 0.3 (Andrich et al., 2012). Compared to, for instance, confirmatory factor analysis, Rasch modeling offers a more comprehensive assessment of the performance quality at the item level, which is appropriate for designing, structure, and evaluating measurement scales (Hambleton et al., 1991).

Independent sample *t-*tests was used to compare the mean score of DHL/DHC between two groups. Using a count as dependent variable, such as health services utilization (number of visits to GP, emergency, and specialist), of which variance significantly exceeds the mean, this study applied negative binomial regression model (NB) instead of Poisson. While the assumption of the Poisson is that variance and the mean is equal, NB allows the variance to be greater than the mean (Hilbe, 2014). Ability to process digital health information (measured by HLS_19_-DIGI) and ability to use DHS (measured by the DHC scale) were used as independent variables. Other sociodemographic covariates such as age, sex, education, social status, and financial deprivation were used as control variables.

Rasch modeling was carried out using the software RUMM2030plus (RUMM Laboratory Pty Ltd., 2019) and ConQuest 5 (Adams et al., 2022). Internal consistency reliability, Omega, was estimated using Mplus version 8.6 (Muthén & Muthén, 1998 to 2017) and Microsoft Excel (for calculating Omega) (Dueber, 2017), while descriptive statistics and regression models were conducted using Stata/SE 16.1 Windows. Statistical significance was set at 5% level across analyses.

### Ethical Consideration

This study was conducted in accordance with the ethical principles of the Declaration of Helsinki. The questionnaire interviews were completed anonymous, and participation was voluntary. As telephone interviews were used, only verbal informed consent was obtained from the participants. This study was outside the scope of the Norwegian Act of Medical and Health Research, and approval from the Norwegian Regional Committees for Medical and Health Research Ethics was not required. However, we notified the Data Protection Services at the Norwegian Centre for Research Data, who has approved the project regarding the use of personal/private data such as questionnaires, consent form, and data storage (project number 896850).

## Results

### Validation of the Digital HealthCare Scale

**Table 1** shows the summary statistics for the Rasch modeling. The person location mean is skewed somewhat toward higher ability to digitally use health care services, which is also reflected in the **Figure A**, showing a ceiling effect, namely that the items may be too easy to endorse among the target population. However, the person separation index (.78) indicated that the DHC item set was quite successful in efficiently separating people at different levels of the scale, and Omega (.94) indicated a good internal consistency. In addition, the DHC scale can be considered sufficiently unidimensional as the proportion of persons with significant different person-location estimates on the compared subscales was barely above 3%. No disordered response categories nor differential item functioning were observed (**Table 2**). The residual correlations for two item pairs are just above 0.3 (item 1/item 6: 0.31 and item 2/item 5: 0.36). As such, this can be considered acceptable concerning response dependence (**Table B**).

**Table 1:**
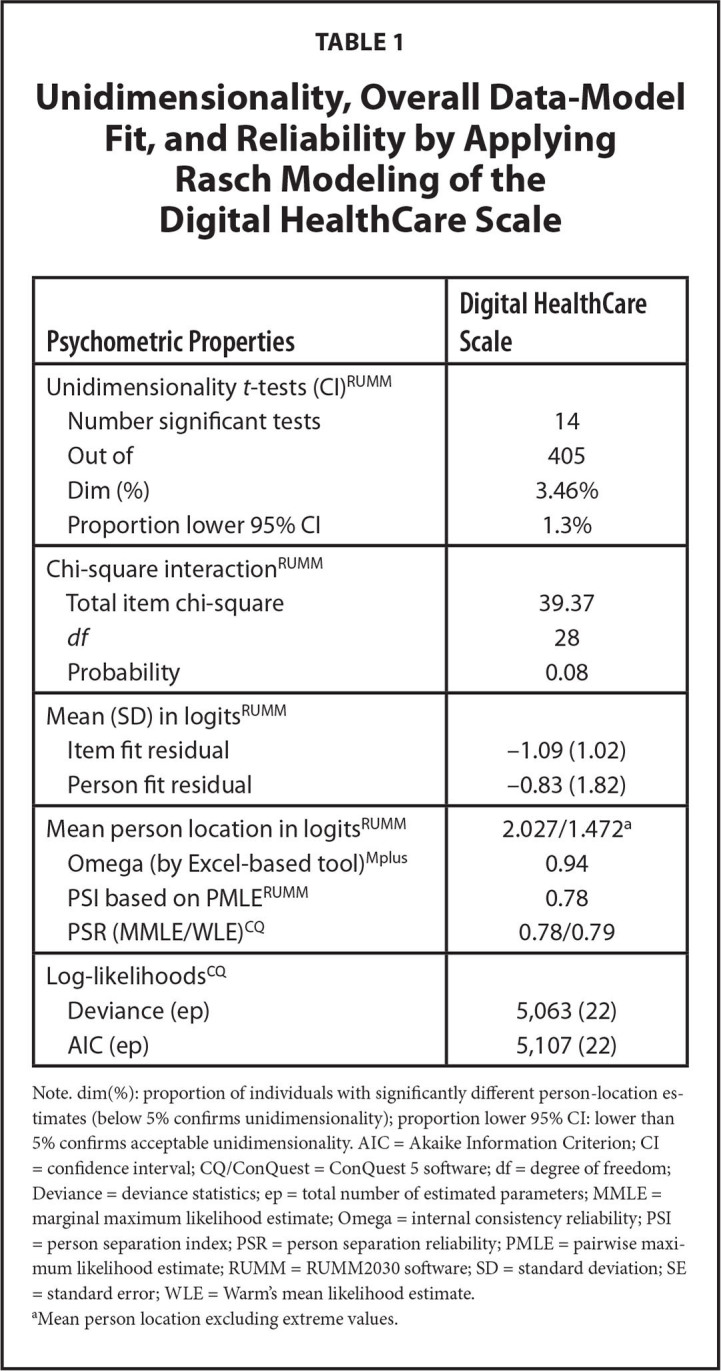
Unidimensionality, Overall Data-Model Fit, and Reliability by Applying Rasch Modeling of the Digital HealthCare Scale

**Psychometric Properties**	**Digital HealthCare Scale**

Unidimensionality *t*-tests (CI)^RUMM^	
Number significant tests	14
Out of	405
Dim (%)	3.46%
Proportion lower 95% CI	1.3%

Chi-square interaction^RUMM^	
Total item chi-square	39.37
*df*	28
Probability	0.08

Mean (SD) in logits^RUMM^	
Item fit residual	−1.09 (1.02)
Person fit residual	−0.83 (1.82)

Mean person location in logits^RUMM^	2.027/1.472^a^
Omega (by Excel-based tool)^Mplus^	0.94
PSI based on PMLE^RUMM^	0.78
PSR (MMLE/WLE)^CQ^	0.78/0.79

Log-likelihoods^CQ^	
Deviance (ep)	5,063 (22)
AIC (ep)	5,107 (22)

Note. dim(%): proportion of individuals with significantly different person-location estimates (below 5% confirms unidimensionality); proportion lower 95% CI: lower than 5% confirms acceptable unidimensionality. AIC = Akaike Information Criterion; CI = confidence interval; CQ/ConQuest = ConQuest 5 software; df = degree of freedom; Deviance = deviance statistics; ep = total number of estimated parameters; MMLE = marginal maximum likelihood estimate; Omega = internal consistency reliability; PSI = person separation index; PSR = person separation reliability; PMLE = pairwise maximum likelihood estimate; RUMM = RUMM2030 software; SD = standard deviation; SE = standard error; WLE = Warm's mean likelihood estimate.

a Mean person location excluding extreme values.

**Figure A.**
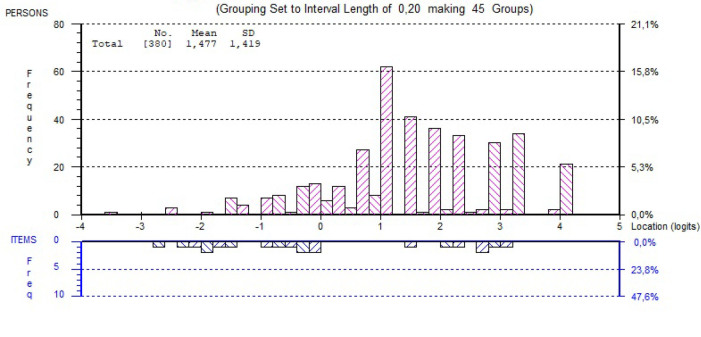
Person-Item Threshold Distribution

**Table 2:**
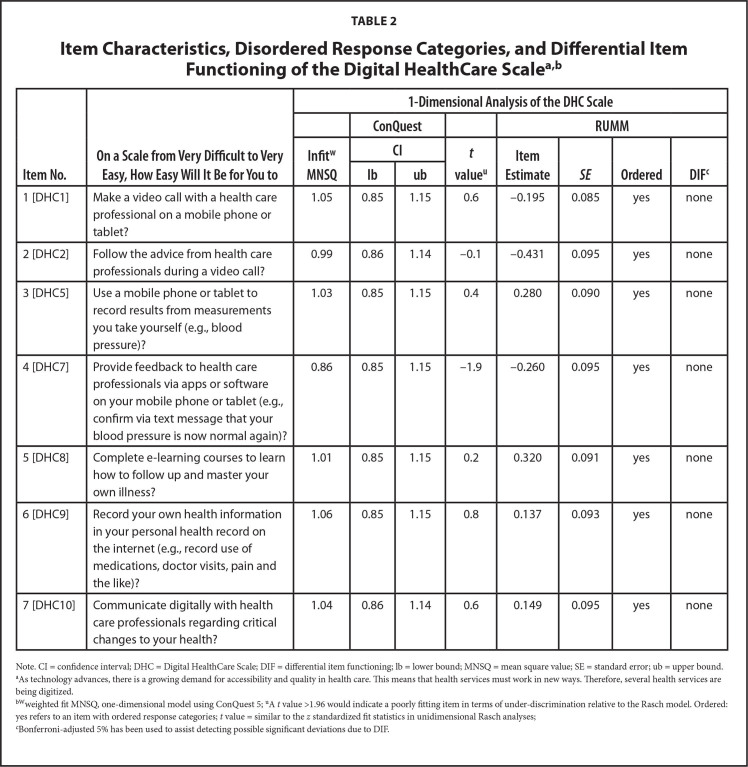
Item Characteristics, Disordered Response Categories, and Differential Item Functioning of the Digital HealthCare Scale^a,b^

**Item No. **	**On a Scale from Very Difficult to Very Easy, How Easy Will It Be for You to**	**1-Dimensional Analysis of the DHC Scale**							
			**ConQuest**			**RUMM**			
		**Infit^w^ MNSQ**	**CI**		***t* value^u^**	**Item Estimate**	***SE***	**Ordered**	**DIF^c^**
			**lb**	**ub**					
1 [DHC1]	Make a video call with a health care professional on a mobile phone or tablet?	1.05	0.85	1.15	0.6	−0.195	0.085	yes	none
2 [DHC2]	Follow the advice from health care professionals during a video call?	0.99	0.86	1.14	−0.1	−0.431	0.095	yes	none
3 [DHC5]	Use a mobile phone or tablet to record results from measurements you take yourself (e.g., blood pressure)?	1.03	0.85	1.15	0.4	0.280	0.090	yes	none
4 [DHC7]	Provide feedback to health care professionals via apps or software on your mobile phone or tablet (e.g., confirm via text message that your blood pressure is now normal again)?	0.86	0.85	1.15	−1.9	−0.260	0.095	yes	none
5 [DHC8]	Complete e-learning courses to learn how to follow up and master your own illness?	1.01	0.85	1.15	0.2	0.320	0.091	yes	none
6 [DHC9]	Record your own health information in your personal health record on the internet (e.g., record use of medications, doctor visits, pain and the like)?	1.06	0.85	1.15	0.8	0.137	0.093	yes	none
7 [DHC10]	Communicate digitally with health care professionals regarding critical changes to your health?	1.04	0.86	1.14	0.6	0.149	0.095	yes	none

Note. CI = confidence interval; DHC = Digital HealthCare Scale; DIF = differential item functioning; lb = lower bound; MNSQ = mean square value; SE = standard error; ub = upper bound.

a As technology advances, there is a growing demand for accessibility and quality in health care. This means that health services must work in new ways. Therefore, several health services are being digitized.

b ^W^weighted fit MNSQ, one-dimensional model using ConQuest 5; A *t* value >1.96 would indicate a poorly fitting item in terms of under-discrimination relative to the Rasch model. Ordered: yes refers to an item with ordered response categories; *t* value = similar to the *z* standardized fit statistics in unidimensional Rasch analyses;

c Bonferroni-adjusted 5% has been used to assist detecting possible significant deviations due to DIF.

**Table B.**
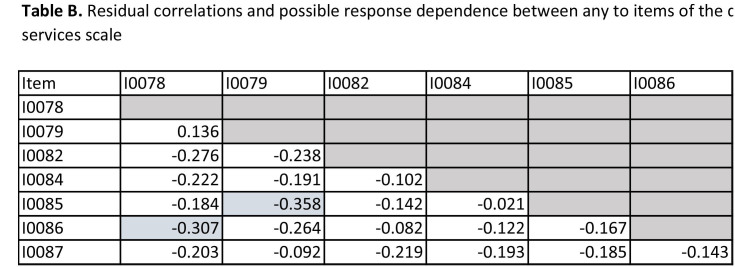
Residual correlations and possible response dependence between any to items of the digital health services scale

Item	I0078	I0079	I0082	I0084	I0085	I0086
I0078						
I0079	0.136					
I0082	−0.276	−0.238				
I0084	−0.222	−0.191	−0.102			
I0085	−0.184	−0.358	−0.142	−0.021		
I0086	−0.307	−0.264	−0.082	−0.122	−0.167	
I0087	−0.203	−0.092	−0.219	−0.193	−0.185	−0.143

### Digital Health Literacy and Ability to Utilize Digital Health Services by Sociodemographic Characteristics

While 890 participants were included in the study (**Table 3**), a smaller sample (*n* = 471) was applied to the DHC scale. There were statistically significant differences in the ability to use DHS using the DHC scale between sex and age groups, in which male participants and those age 21 to 25 years having significantly better abilities than female participants and adolescents age 16 to 20 years, respectively. Adolescents who reported education beyond upper secondary level had a mean score approximately 0.4 logits higher than those with lower education, even no statistical significance was observed. Almost same pattern was observed for DHL using the HLS_19_-DIGI scale, except for sex that was on the borderline of statistical non-significance (*p* = .059). In addition, statistically significant differences were found in the ability to process digital health information according to respondents' financial situation, while self-perceived social status was also on the borderline of statistical non-significance (*p* = .060).

**Table 3:**
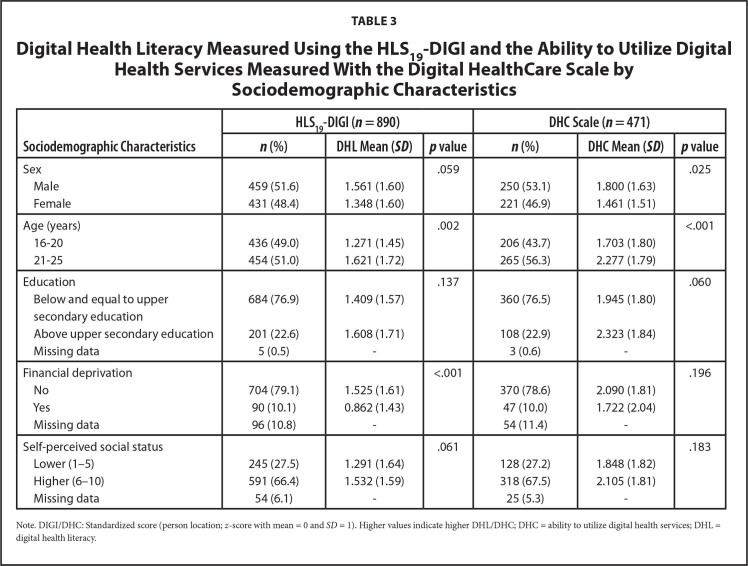
Digital Health Literacy Measured Using the HLS_19_-DIGI and the Ability to Utilize Digital Health Services Measured With the Digital HealthCare Scale by Sociodemographic Characteristics

**Sociodemographic Characteristics**	**HLS_19_-DIGI (*n* = 890)**			**DHC Scale (*n* = 471)**		
	***n* (%)**	**DHL Mean (*SD*)**	***p* value**	***n* (%)**	**DHC Mean (*SD*)**	***p* value**
Sex			.059			.025
Male	459 (51.6)	1.561 (1.60)		250 (53.1)	1.800 (1.63)	
Female	431 (48.4)	1.348 (1.60)		221 (46.9)	1.461 (1.51)	

Age (years)			.002			<.001
16–20	436 (49.0)	1.271 (1.45)		206 (43.7)	1.703 (1.80)	
21–25	454 (51.0)	1.621 (1.72)		265 (56.3)	2.277 (1.79)	

Education			.137			.060
Below and equal to upper	684 (76.9)	1.409 (1.57)		360 (76.5)	1.945 (1.80)	
secondary education						
Above upper secondary education	201 (22.6)	1.608 (1.71)		108 (22.9)	2.323 (1.84)	
Missing data	5 (0.5)	-		3 (0.6)	-	

Financial deprivation			<.001			.196
No	704 (79.1)	1.525 (1.61)		370 (78.6)	2.090 (1.81)	
Yes	90 (10.1)	0.862 (1.43)		47 (10.0)	1.722 (2.04)	
Missing data	96 (10.8)	-		54 (11.4)	-	

Self-perceived social status			.061			.183
Lower (1–5)	245 (27.5)	1.291 (1.64)		128 (27.2)	1.848 (1.82)	
Higher (6–10)	591 (66.4)	1.532 (1.59)		318 (67.5)	2.105 (1.81)	
Missing data	54 (6.1)	-		25 (5.3)	-	

Note. DIGI/DHC: Standardized score (person location; *z*-score with mean = 0 and *SD* = 1). Higher values indicate higher DHL/DHC; DHC = ability to utilize digital health services; DHL = digital health literacy.

### Use of Health Services Among Adolescents by Dual Digital Ability

Adjusted for age, sex, education, self-perceived social status, and financial situation, **Table 4** and **Table 5** show that DHL (incidence-rate ratio [IRR] = .75, *p* = .003, 95% confidence interval [CI]: .62 to .91) and DHS (I*RR* = .72, *p* < .001, 95% CI: .61 to .84) were associated with the utilization of specialists. The IRR indicates that for every unit (logit) increase in DHL and DHC, the number of specialist visits decreases by 25% and 28%, respectively.

**Table 4:**
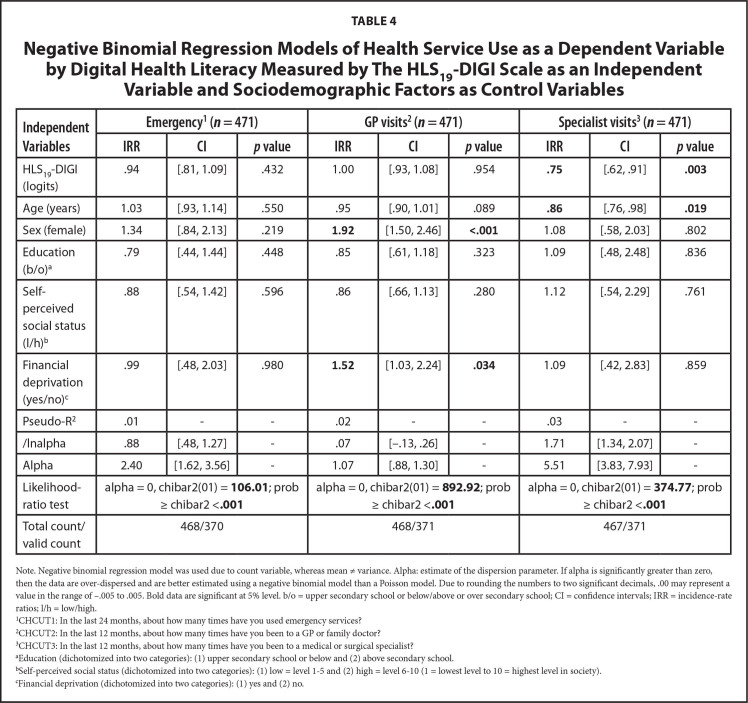
Negative Binomial Regression Models of Health Service Use as a Dependent Variable by Digital Health Literacy Measured by The HLS_19_-DIGI Scale as an Independent Variable and Sociodemographic Factors as Control Variables

**Independent Variables**	**Emergency^1^ (*n* = 471)**			**GP visits^2^ (*n* = 471)**			**Specialist visits^3^ (*n* = 471)**		
	**IRR**	**CI**	***p* value**	**IRR**	**CI**	***p* value**	**IRR**	**CI**	***p* value**
HLS_19_-DIGI (logits)	.94	[.81, 1.09]	.432	1.00	[.93, 1.08]	.954	**.75**	[.62, .91]	**.003**
Age (years)	1.03	[.93, 1.14]	.550	.95	[.90, 1.01]	.089	**.86**	[.76, .98]	**.019**
Sex (female)	1.34	[.84, 2.13]	.219	**1.92**	[1.50, 2.46]	**<.001**	1.08	[.58, 2.03]	.802
Education (b/o)^a^	.79	[.44, 1.44]	.448	.85	[.61, 1.18]	.323	1.09	[.48, 2.48]	.836
Self-perceived social status (l/h)^b^	.88	[.54, 1.42]	.596	.86	[.66, 1.13]	.280	1.12	[.54, 2.29]	.761
Financial deprivation (yes/no)^c^	.99	[.48, 2.03]	.980	**1.52**	[1.03, 2.24]	**.034**	1.09	[.42, 2.83]	.859
Pseudo-R^2^	.01	-	-	.02	-	-	.03	-	-
/lnalpha	.88	[.48, 1.27]	-	.07	[−.13, .26]	-	1.71	[1.34, 2.07]	-
Alpha	2.40	[1.62, 3.56]	-	1.07	[.88, 1.30]	-	5.51	[3.83, 7.93]	-
Likelihood-ratio test	alpha = 0, chibar2(01) = **106.01**; prob ≥ chibar2 <**.001**			alpha = 0, chibar2(01) = **892.92**; prob ≥ chibar2 <**.001**			alpha = 0, chibar2(01) = **374.77**; prob ≥ chibar2 <**.001**		
Total count/valid count	468/370			468/371			467/371		

Note. Negative binomial regression model was used due to count variable, whereas mean ≠ variance. Alpha: estimate of the dispersion parameter. If alpha is significantly greater than zero, then the data are over-dispersed and are better estimated using a negative binomial model than a Poisson model. Due to rounding the numbers to two significant decimals, .00 may represent a value in the range of −.005 to .005. Bold data are significant at 5% level. b/o = upper secondary school or below/above or over secondary school; CI = confidence intervals; IRR = incidence-rate ratios; l/h = low/high.

1 CHCUT1: In the last 24 months, about how many times have you used emergency services?

2 CHCUT2: In the last 12 months, about how many times have you been to a GP or family doctor?

3 CHCUT3: In the last 12 months, about how many times have you been to a medical or surgical specialist?

a Education (dichotomized into two categories): (1) upper secondary school or below and (2) above secondary school.

b Self-perceived social status (dichotomized into two categories): (1) low = level 1–5 and (2) high = level 6–10 (1 = lowest level to 10 = highest level in society).

c Financial deprivation (dichotomized into two categories): (1) yes and (2) no.

**Table 5:**
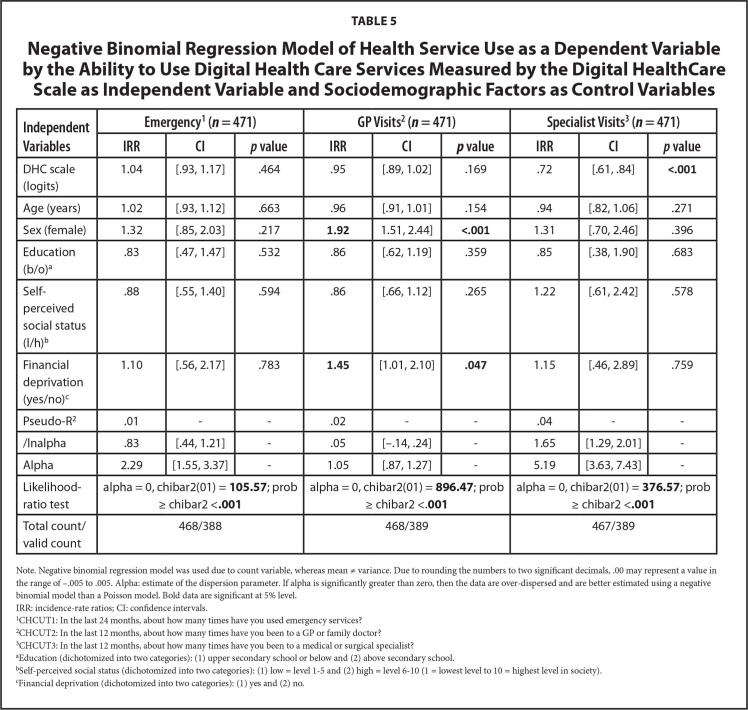
Negative Binomial Regression Model of Health Service Use as a Dependent Variable by the Ability to Use Digital Health Care Services Measured by the Digital HealthCare Scale as Independent Variable and Sociodemographic Factors as Control Variables

**Independent Variables**	**Emergency^1^ (*n* = 471)**			**GP Visits^2^ (*n* = 471)**			**Specialist Visits^3^ (*n* = 471)**		
	**IRR**	**CI**	***p* value**	**IRR**	**CI**	***p* value**	**IRR**	**CI**	***p* value**
DHC scale (logits)	1.04	[.93, 1.17]	.464	.95	[.89, 1.02]	.169	.72	[.61, .84]	**<.001**
Age (years)	1.02	[.93, 1.12]	.663	.96	[.91, 1.01]	.154	.94	[.82, 1.06]	.271
Sex (female)	1.32	[.85, 2.03]	.217	**1.92**	1.51, 2.44]	**<.001**	1.31	[.70, 2.46]	.396
Education (b/o)^a^	.83	[.47, 1.47]	.532	.86	[.62, 1.19]	.359	.85	[.38, 1.90]	.683
Self-perceived social status (l/h)^b^	.88	[.55, 1.40]	.594	.86	[.66, 1.12]	.265	1.22	[.61, 2.42]	.578
Financial deprivation (yes/no)^c^	1.10	[.56, 2.17]	.783	**1.45**	[1.01, 2.10]	**.047**	1.15	[.46, 2.89]	.759
Pseudo-R^2^	.01	-	-	.02	-	-	.04	-	-
/lnalpha	.83	[.44, 1.21]	-	.05	[–.14, .24]	-	1.65	[1.29, 2.01]	-
Alpha	2.29	[1.55, 3.37]	-	1.05	[.87, 1.27]	-	5.19	[3.63, 7.43]	-
Likelihood-ratio test	alpha = 0, chibar2(01) = **105.57**; prob ≥ chibar2 <**.001**			alpha = 0, chibar2(01) = **896.47**; prob ≥ chibar2 <.**001**			alpha = 0, chibar2(01) = **376.57**; prob ≥ chibar2 <**.001**		
Total count/valid count	468/388			468/389			467/389		

Note. Negative binomial regression model was used due to count variable, whereas mean ≠ variance. Due to rounding the numbers to two significant decimals, .00 may represent a value in the range of −.005 to .005. Alpha: estimate of the dispersion parameter. If alpha is significantly greater than zero, then the data are over-dispersed and are better estimated using a negative binomial model than a Poisson model. Bold data are significant at 5% level.

IRR: incidence-rate ratios; CI: confidence intervals.

1 CHCUT1: In the last 24 months, about how many times have you used emergency services?

2 CHCUT2: In the last 12 months, about how many times have you been to a GP or family doctor?

3 CHCUT3: In the last 12 months, about how many times have you been to a medical or surgical specialist?

a Education (dichotomized into two categories): (1) upper secondary school or below and (2) above secondary school.

b Self-perceived social status (dichotomized into two categories): (1) low = level 1–5 and (2) high = level 6–10 (1 = lowest level to 10 = highest level in society).

c Financial deprivation (dichotomized into two categories): (1) yes and (2) no.

## Discussion

### Psychometric Properties of the Digital HealthCare Scale

The DHC data displayed sufficiently overall fit to the partial credit model by means of non-significant overall chi-square statistic and the scale seemed to be unidimensional. No problems at the item level were observed, whereas both infit and T-values seemed acceptable. The scale however obtained a relatively high value of mean person location, and the ceiling effect have been witnessed by extreme person scores. Nonetheless, the ordering of the response categories remains good, as well as there is no differential item functioning. The phenomenon of high mean person location is somehow expected in the target population as they are more digital in nature than the older population (Keles et al., 2020; Lenhart, 2015a; Lenhart, 2015b). However, poor targeting due to a high value of mean person location may have resulted in deflated variance in person estimates. This could further have led to poor person separation and deflated reliability indexes (Tennant & Conaghan, 2007). When removing the extreme person scores, the person-item threshold seemed more normally distributed (**Figure A)**, but the targeting could be better. Hence, the scale could benefit from adding more or other items that are harder to endorse.

### Digital Health Literacy and Ability to Utilize Digital Health Services Among Adolescents

Previous studies revealed that most adolescents are active on social media, and those specifically in the age range between 13- to 17-year-old were identified as particularly heavy users of social media using computer, tablet device, or smartphone (Keles et al., 2020; Lenhart, 2015a; Lenhart, 2015b). Of concern, our study suggests that female participants and adolescents age 16 to 20 years have significantly lower DHL in relation to male participants and young adults age 21 to 25 years, independent of the education level. This may have resulted in lower DHC in adolescents compared to young adults. Further research may explore this relationship more closely. However, this finding could be interpreted in light of previous research revealing that social media use is considered a positive leisure activity for male youth while having negative impact on female youth (Blomfield Neira & Barber, 2014).

### The Associations Between Digital Health Literacy/Digital HealthCare and Health Service Use

Having controlled for sociodemographic factors such as age, sex, education, self-perceived social status, and self-reported financial deprivation, DHL and the ability to use DHS still affected the extent to which adolescents use specialist health services. However, there was neither association between DHL/DHC and primary health care use in terms of GP nor emergency service visits. This finding may be explained by the fact that digital services are offered to a lesser extent in the primary health care (GP/emergency services) than in specialist care, due to the slower pace of digitalization. Knowing that DHL increases with age, it is important to pay attention to the fact that health problems, the need for disease prevention, and the development of health promoting behavior may occur any time during childhood and adolescence (Blakemore & Mills, 2014; Brumbach et al., 2009; Green et al., 2013; Kessler et al., 2005; Whitaker et al., 2013). As well, they use health promotion and disease prevention services to a lesser extent than adults (Callahan & Cooper, 2010; Fortuna et al., 2009). The latter may be due to a lack of (digital) health literacy and ability to use DHS. Furthermore, the finding that aging is not associated with increased ability to use DHS has strengthened the case that calls for more efforts to improve adolescents' ability to use DHS as long as digitalization of health services keeps expanding and accelerating.

## Study Strengths and Limitations

The study's novelty is a strength; to our knowledge, no unidimensional instrument exists that measures people's ability to use DHS, especially for adolescents and young adults. Although the study sample was population-based using representative strata and highly comparable with Statistics Norway (2024) concerning the distribution of age and sex, which is a strength, the measurement instrument should be tested using data from multiple countries. This instrument should be tested in people with long-term illness and in older adults who might be more dependent on digital health follow up.

The sample size (*n* = 471) used to assess the psychometric properties of the DHC scale could have been larger even though it is not strictly required when using Rasch modeling. Furthermore, the content validity and understandability of the item set could have been tested using cognitive interviews with representatives from the target population. However, the scale had already been piloted among a random sample from several institutions and organizations such as directorates, municipalities, universities, and non-governmental organizations. Based on empirical observations from the pilot study and the interpretation in light of theoretical expectations, amendments and adaptation of the item set were made prior to the main data collection (Le et al., 2021).

## Conclusion

The DHC scale is considered a valid unidimensional scale measuring the ability to use DHS in young people age 16 to 25 years. This instrument is likely to be a useful measure for health authorities in their processes toward planning, developing, implementing, and evaluating DHS. Empirical evidence from the study may provide new knowledge-based insights that are essential for improving young people's ability to use DHS, and eventually preventing unnecessary use of health services, especially specialist health care. Cross-sectional studies are not suitable for drawing conclusions about the effect of a measure, so the results of the study should be interpreted with cautions. To establish the DHC scale's generalized validity, further research using samples from different countries and subpopulations are warranted.
